# A New Microscope

**Published:** 1877-09

**Authors:** 


### EDITORIAL.

### A NEW MICROSCOPE.

The microscope has come to be an instrument of very great importance, in almost every avocation of life. It opens up fields for observation and study, that without it, would have remained hidden and sealed; its revealments are interesting to all, and instructive and profitable to many.

Every member of any learned profession should be familiar with the microscope and its use.

He who engages in any department of science finds in the microscope an auxiliary that he cannot afford to be without; the artist too will find in it a valuable assistant.

The desire to look further and further into nature is a growing one, and cannot be adequately met without the aid which the microscope only can give.

If these things are true, why is it, that this instrument is employed to so limited an extent? An answer to this question is found in the fact that the instrument and its accessories have been hitherto so expensive that but few have felt warranted in making the outlay requisite to secure a good microscope.

This disability to some extent at least, has been removed by Messrs. Bausch & Lomb, of New York, and also of Rochester, New York.

The instruments made by this firm are equal to any we have seen: the mechanism is equal to the best and the workmanship unexceptionable.

The objectives are very good indeed, quite sufficient for all ordinary work, and all the other accessories are equal to those attached to far more expensive instruments.

The idea of these gentlemen to popularize and bring the microscope within the reach of a far greater number of persons is a grand one. The effort should be encouraged.

A good microscope is now within the pecuniary reach of almost every one.

